# Occurrence of cavernicolous ground beetles in Anhui Province, eastern China (Coleoptera, Carabidae, Trechinae)

**DOI:** 10.3897/zookeys.625.9846

**Published:** 2016-10-19

**Authors:** Jie Fang, Wenbo Li, Mingyi Tian

**Affiliations:** 1 College of Life Science, Anhui University, Hefei, 230039, China; 2 Department of Entomology, College of Agriculture, South China Agricultural University, 483 Wushan Road, Guangzhou, 510642, China

**Keywords:** Carabid beetle, new genus, new species, troglobitic

## Abstract

Two new species of anophthalmic ground beetles belonging to the subfamily Trechinae are described: Cimmeritodes (Zhecimmerites) parvus Tian & Li, **sp. n.** and *Wanoblemus wui* Tian & Fang, **gen. n., sp. n.** Both were discovered in the limestone caves of Anhui Province in eastern China. Cimmeritodes (Zhecimmerites) parvus was found in caves Ziwei Dong, Xianren Dong and Qingtai Dong, whereas *Wanoblemus wui* was discovered in cave Baiyun Dong. This is the first record of cavernicolous ground beetles in Anhui Province, eastern China.

## Introduction

Trechine beetles are the most speciose group of insects in cave fauna (Moldovan 2012) and are distributed across every continent (Casale et al. 1998). Within the tribe Trechini, the phyletic series (Jeannel 1928; Casale and Laneyrie 1982; Casale et al. 1998) or complex (Uéno and Pawlowski 1981) *Trechoblemus* appears to be among the most diverse groups, comprising about 300 known species belonging to 16 genera. The first genus of this series described in China, *Cimmeritodes*,﻿ is represented by a single species endemic to the Hunan Province (Deuve 1996). The genera *Wulongoblemus* and *Microblemus* were newly described in the Zhejiang Province (Uéno 1997), whereas the endemic genera *Balazucellus* and *Sinocimmerites* were described from the Hubei and Fujian Provinces, respectively (Deuve 2001, 2007). More recently, Tian and Yin (2013) reported *Sidublemus* from the Hunan Province and Deuve and Tian (2015) described the new subgenus Zhecimmerites (Cimmeritodes) from eastern Zhejiang.

More than 600 caves are located in Anhui Province (eastern China), the majority of which spans both banks of the Yangtze River (Lu 2012). Unlike the karstic landscape characteristic of southern China provinces, the limestone landforms of Anhui Province are regularly distributed. Their geographic scale and relevant topographic features are primarily influenced by stratum’s lithology and structure, as well as by hydrogeological conditions (Sun et al. 2000). Despite the presence of suitable cavernicolous environments, no trechine ground beetles have been recorded in Anhui so far (Tian et al. 2016).

In 2015, 11 anophthalmic trechine beetles were collected from caves in the Chaohu–Wuwei–Xuancheng karstic areas, as part of a series of biological surveys conducted in Anhui’s caves. Further studies confirmed that these beetles belonged to two taxa not recorded in this Province: the subgenus *Zhecimmerites* (genus *Cimmeritodes*) and the new genus *Wanoblemus* gen. n., which is proposed in this paper. This is the first report of cavernicolous trechine beetles in Anhui Province.

## Material and methods

Specimens were collected in cave by aspiration and kept in 55% ethanol until their dissection and observation under a Leica S8AP0 stereomicroscope. Dissected genital pieces, including the median lobe and the parameres of the aedeagus, were glued onto small transparent plastic plates and pinned beneath the specimen to which they belonged. Habitus were photographed using a Keyence VHX-5000 digital microscope and the genital pieces were photographed using a Canon EOS 40D camera connected to a Zeiss AX10 microscope. Photographs were then stacked and processed using Adobe Photoshop CS5 software. Distribution maps were obtained in MapInfo.

Body length was measured from the apex of the right mandible (in the open position) to the elytral apex; body width corresponded to the maximum width of the elytra.

Abbreviations of other measurements are as follows, following Tian et al. (2016):

HLm
 head length including mandibles, measured from the apex of the right mandible to the occipital suture 


HLl
 head length excluding mandibles, measured from the front of the labrum to the occipital suture 


HW
 maximum head width 


PnL
 pronotum length along the median line 


PnW
 maximum pronotum width 


PfW
 pronotum width at front 


PbW
 pronotum width at base 


EL
 elytra length, from base of scutellum to elytral apex 


EW
 maximum width of combined elytra 


## Taxonomy

### Cimmeritodes (Zhecimmerites) parvus

Taxon classificationAnimaliaColeopteraCarabidae

Tian & Li sp. n.

http://zoobank.org/F0002E70-E282-4E32-8D43-90393C99344A

Figs 1 , 2 , 3

#### Material.

Holotype, male, Ziwei Dong (also called Shuangjing Dong) Cave, Chaohu Shi, 31.6479N, 117.8632E, 83 m altitude, IV-21-2015, leg. Yunhe Wu and Wenbo Li, deposited in the insect collections of South China Agricultural University
(SCAU), Guangzhou. Paratypes: one male and three females, ibid., deposited in SCAU and the animal collections of Anhui University
(ANU), Hefei, respectively.

#### Additional material.

One male, Cave Xianren Dong, suburbs of southern Chaohu Shi, 31.4633N, 117.8413E, 110 m altitude, IV-27-2015, leg. Yunhe Wu and Wenbo Li, deposited in SCAU; one female, Boshan Dong Cave, Xiaboshan, 38 km SW of the main town of Wuwei County, 31.1851N, 117.5582E, 28 m altitude, IV-28-2015, leg. Yunhe Wu and Wenbo Li, deposited in SCAU; two males, Qingtai Dong Cave, Qingtaishan, NW Shijian Zhen, Wuwei County, 31.5330N, 117.0244E, 79 m altitude, IV-22-2015, leg. Yunhe Wu and Wenbo Li, deposited in SCAU; one female, Boshan Dong Cave, Xiaboshan, Shushan Zhen, Wuwei County, 31.1851N, 117.5582E, 28 m altitude, IV-28-2015, leg. Yunhe Wu and Wenbo Li, in SCAU.

#### Diagnosis.

Small-sized, anophthalmic trechine beetles, with yellowish brown and stout body, short antennae and legs, fore body distinctly shorter than elytra.

#### Description.

**Figure 1.**
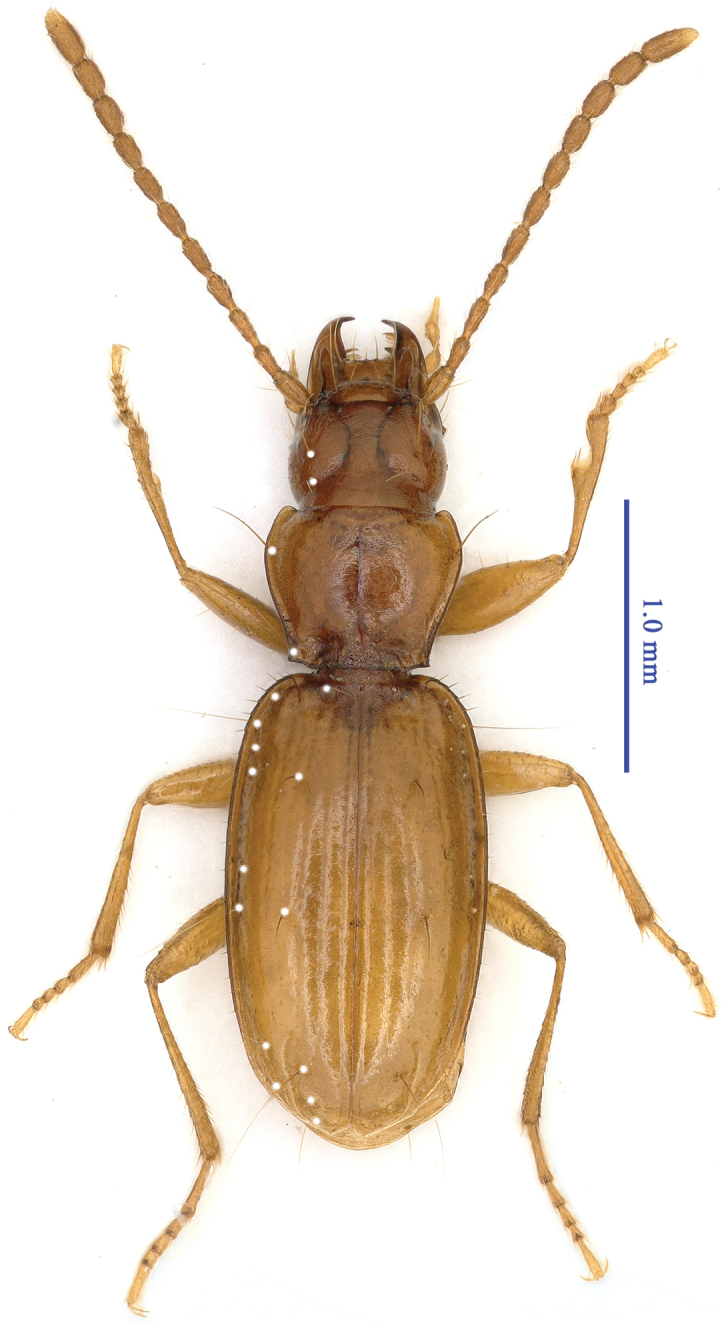
Habitus of Cimmeritodes (Zhecimmerites) parvus Tian & Li, sp. n., female, paratype.

Length: 3.0–3.7 mm, including mandibles (or 2.8–3.5 mm, excluding mandibles); width: 1.0 mm. Habitus as in Fig. 1.

Small-sized trechine species. Body yellowish brown and palps, antennae, and tarsi pale; moderately shiny; finely pubescent though frons; vertex, ventral head, and prosternum glabrous; micro sculptural meshes moderately isodiametric on head, moderately transverse on pronotum, and strongly transverse on elytra. Mandibles much shorter than elytra, EL/(HLm + PnL) = 2.31, in the front part of the body

HLm/HW = 1.22, HLl/HW = 0.94, right mandible tridentate, median mandible reduced; labial suture absent; submentum 6-setose; mentum bisetose, without basal pits; tooth short, bifid at apex; palps short and stout; labial palpomere 2 longer than 3, which is very thin, bisetose on inner margin, with two or three additional setae at the subapex; palpomere 3 much more slender than 2; maxillary palpomeres 3 and 4 subequal in length; ligula short and rounded at apex, 8-setose. Antennae short, extending to the middle of the elytra.

Pronotum transverse, PnL/PnW = 0.83, wider than the head, PnW/HW = 1.24, shorter than the head (including mandibles), HLm/PnL = 1.19, or as long as the head (excluding mandibles); disc moderately convex; pronotum widest at about 1/3 from the apex, fore and hind lateromarginal setae located just before the widest point and the hind angle, respectively; fore angles rounded, basal angles sharp; pronotum base narrower than front, PfW/PbW = 1.12; front almost straight, base nearly straight medially, obtusely sinuate near hind angles. Scutellum small and short.

Elytra elongate, thin, moderately convex, much longer than wide, EL/EW =1.74, sides ciliated, gently expanded laterally, widest near the middle, evenly narrowed towards the base and the subapex; elytra base wide, shoulders rounded; disc moderately convex, deeply striated, apical striae well-marked, intervals slightly convex; basal pore present; anterior dorsal pore located on 3^rd^ stria at about 2/9 of the base, middle pore on the 4^th^ interval, a little behind the middle of the elytra; pre-apical pore located exactly at the end of the 4^th^ stria, at about 1/7 of elytra apex, subequal to the apex and to the suture; humeral group of marginal umbilicate series equidistant, both pores of the middle group closely located.

Legs moderately long, covered with dense and short hairs; protarsi short, 1^st^ tarsomere slightly wider than the others, as long as the 2^nd^ and 3^rd^ combined, 4^th^ protarsomere as long as wide; 1^st^ tarsomere as long as, or longer than 2^nd^ – 4^th^ tarsomere combined in meso- and metatarsi, respectively. Ventrites IV–VI bearing a pair of paramedian setae; ventrite VII 4-setose in female, but bisetose in male.

**Figure 2.**
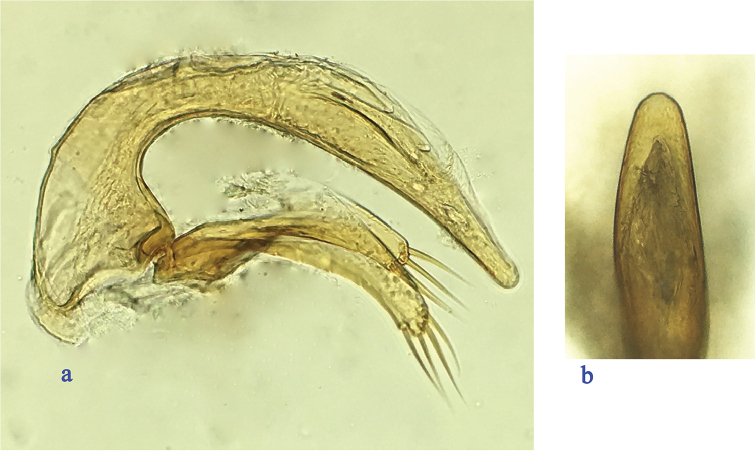
Male genitalia of Cimmeritodes (Zhecimmerites) parvus Tian & Li, sp. n., **a** median lobe and parameres, lateral view **b** apical lobe, dorsal view.

Male genitalia (Fig. 2): median lobe of the aedeagus well-sclerotized, small and thin, strongly curved ventrally in the middle, gradually narrowed towards the apex, which is blunt; base moderately large, sagittal aileron small and thin, inner sac provided with a short and very thin copulatory piece, which is about one fifth of the aedeagus in length; in dorsal view, apical lobe short and broad; parameres elongate and thin, subequal, each armed with four (right paramere) and three (left paramere) long setae at apex.

#### Remarks.

Similar to Cimmeritodes (Zhecimmerites) zhejiangensis Deuve & Tian, 2015, which occurs in eastern Zhejiang Province, but with different male genital structures. Specifically, in Cimmeritodes (Zhecimmerites) parvus, the median lobe is distinctly curved, and shorter and stouter than in Cimmeritodes (Zhecimmerites) zhejiangensis.

#### Etymology.

Indicates the small-sized body of this new species.

#### Distribution.

**Figure 3.**
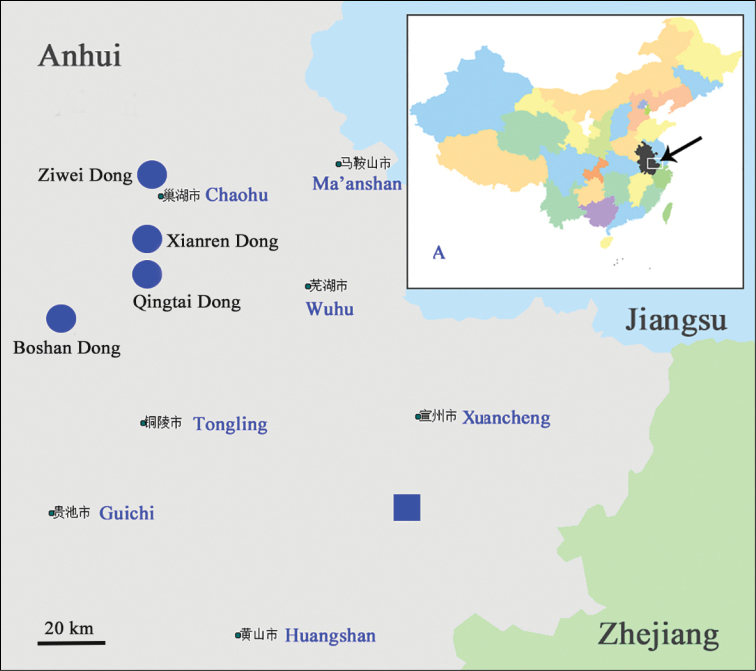
Distribution map of cave-dwelling trechines in Anhui Province **A** map of China, arrowhead showing the cave biological study area; circle localities of Cimmeritodes (Zhecimmerites) parvus Tian & Li, sp. n., square localities of *Wanoblemus wui* Tian & Fang, gen. n., sp. n.

China (Anhui) (Fig. 3). Xianren Dong Cave is a well-developed cave, approximately 450 m in length. Numerous stalactites and white peonies are found within the cave. Ziwei Dong Cave is a typical subterranean river cave, more than 3,000 m long. It is a show cave with uneven ground and numerous stalactites. Boshan Dong Cave is approximately 500 m long and has an uneven and complex tunnel. It is also a show cave, containing stone flowers and waterfalls. Qingtai Dong Cave is approximately 4,000 m in length, has a deep and long tunnel and a unique and outstanding interior. Beetle specimens were sampled from the ground surfaces of the caves, which were covered with litter and/or bat guano.

### Wanoblemus

Taxon classificationAnimaliaColeopteraCarabidae

Tian & Fang gen. n.

http://zoobank.org/15A4B7B5-33B0-4AF9-8951-7248849F46D7

#### Type species.

*Wanoblemus wui* Tian & Fang, sp. n. (Baiyun Dong Cave, Xuancheng Shi).

#### Generic characteristics.

Medium-sized within the phyletic series (Jeannel 1928; Casale and Laneyrie 1982; Casale et al. 1998) or complex (Uéno and Pawlowski 1981) *Trechoblemus*; anophthalmic beetles; body and appendages fairly thin and elongate; head subquadrate, longer (excluding mandibles) than wide, and longer than pronotum; frontal furrows complete, two pairs of supra-orbital and one pair of suborbital pores present; right mandible bidentate; labial suture completely absent, resulting in fused mentum and submentum; mentum bisetose, base strongly concave, tooth simple and short, blunt at apex; submentum 10-setose; antennae fairly long, nearly reaching the middle of the elytra; pronotum subcordate, slightly shorter than wide, widest near the front, about 1/5 of the apex, with two pairs of lateromarginal setae; lateral margin of the pronotum just before the hind angles nearly parallel, hind angles rectangular and sharp, the base nearly straight; elytra elongate, much longer (including mandibles) than the fore body, widest near the middle, surface moderately convex, shoulders distinct and angularly rounded where finely subserrate; lateral margins ciliated throughout; striae obliterated in stria 1, partly traceable in striae 2 and 3; two dorsal and the pre-apical pores present on each elytron; humeral group of marginal umbilicate pores irregular; protibia with external longitudinal groove; in males, protarsomeres 1 and 2 modified, distinctly denticulate inward, at the apex; ventrite VII with two pairs of setae in females and one pair of setae in males; male genitalia short and stout, strongly curved.

#### Discussion.

*Wanoblemus* is not clearly related to any trechine genus associated with the *Trechoblemus* phyletic series described in China. It is probably closest to the Zhejiangese genus *Wulongoblemus* Uéno, 2006, as both have a similar pronotum and modified protarsomeres 1 and 2 in males. However, *Wanoblemus* differs from *Wulongoblemus* in the following characteristics: (1) right mandible bidentate in *Wanoblemus* but tridentate in *Wulongoblemus*; (2) elytra not serrate in the shoulders, less convex and narrower in *Wanoblemus* than in *Wulongoblemus*; (3) *Wanoblemus* is smaller than *Wulongoblemus*; (4) in *Wanoblemus* the pronotum is covered with fairly long pubescence, hind angles are right, postangular carinae are indistinct, and the lateral margin before hind angles is straight and nearly parallel, whereas in *Wulongoblemus* the pronotum is glabrous, hind angles are sharp, postangular carinae are distinct, and the lateral margin before hind angles is strongly sinuate, not parallel.

*Wanoblemus* might also be closely related to the subgenus *Zhecimmerites* (genus *Cimmeritodes*), although differing from the latter in the following key characteristics: (1) members of *Wanoblemus* are larger, and their body and appendages are more elongated than that of members of the genus *Cimmeritodes*; (2) the right mandible is bidentate in *Wanoblemus* and tridentate in *Cimmeritodes*; (3) in male *Wanoblemus* protarsomeres 1 and 2 are modified whereas in male *Cimmeritodes* only protarsomere 1 is modified; (4) *Wanoblemus* pronotum is as long as wide, with right hind angles whereas *Cimmeritodes* pronotum is transverse, with acute hind angles.

A number of characteristics also differ between *Wanoblemus* and the Zhejiangese genus *Microblemus* Uéno, 2007: (1) head quadrate in *Wanoblemus*, but not quadrate in *Microblemus*; (2) right mandible bisetose in *Wanoblemus* and 3-setose in *Microblemus*; (3) mentum and submentum completely fused in *Wanoblemus* and only partly fused, with labial suture traceable, in *Microblemus* ; (4) base of pronotum nearly straight in *Wanoblemus*, with right hind angles, and distinctly emarginated at the median section, with obtuse hind angles, in *Microblemus*; (5) elytra shoulders not dentate in *Wanoblemus* but remarkably dentate in *Microblemus*.

*Wanoblemus* is also easily separated from *Sidublemus* Tian & Yin, 2013, which is found in the southeast of the Hunan Province. In both genera, males protarsi are modified in joints 1 and 2, but: (1) *Wanoblemus* body is larger and more elongated, with slender appendages than *Sidublemus* body, which is small but stout, with short appendages; (2) right mandible bidentate in *Wanoblemus* and tridentate in *Sidublemus*; (3) simple hind angle in *Wanoblemus* and dentate in *Sidublemus*.

*Wanoblemus* is clearly distinct from the genus *Sinocimmerites* due to its elongate body, long and thin appendages, completely fused mentum and submentum, simple head tooth, longitudinally furrowed protibia, and stout and short aedeagus.

The endogean genus *Balazucellus*, which was recorded from Shennongjia, western Hubei Province, also differs from *Wanoblemus*. Among other features, *Balazucellus* body is smaller and stouter than *Wanoblemus*, the right mandible is tridentate (bidentate in *Wanoblemus*), and the antennae are moniliform (filiform in *Wanoblemus*).

#### Etymology.

Indicates these beetles occur in Anhui Province; “Wan” is the short name for Anhui Province in Chinese.

#### Genus distribution range.

China (Anhui) (Fig. 3).

### Wanoblemus wui

Taxon classificationAnimaliaColeopteraCarabidae

Tian & Fang sp. n.

http://zoobank.org/5C9EF8D0-0D00-4C8B-880F-AC7685908D80

#### Material.

Holotype: male, Baiyun Dong Cave, Huayang Xiang, Xuancheng, 30.3737N, 118.4457E, 300 m altitude, X-25-2015, leg. Yunhe Wu and Wenbo Li, deposited in SCAU. Paratypes: one male and one female, ibid., deposited in SCAU and ANU, respectively.

#### Diagnosis.

Medium-sized *Trechoblemus* beetles, with brownish, sparsely pubescent and elongated body, and rather short and stout appendages.

#### Description.

**Figure 4.**
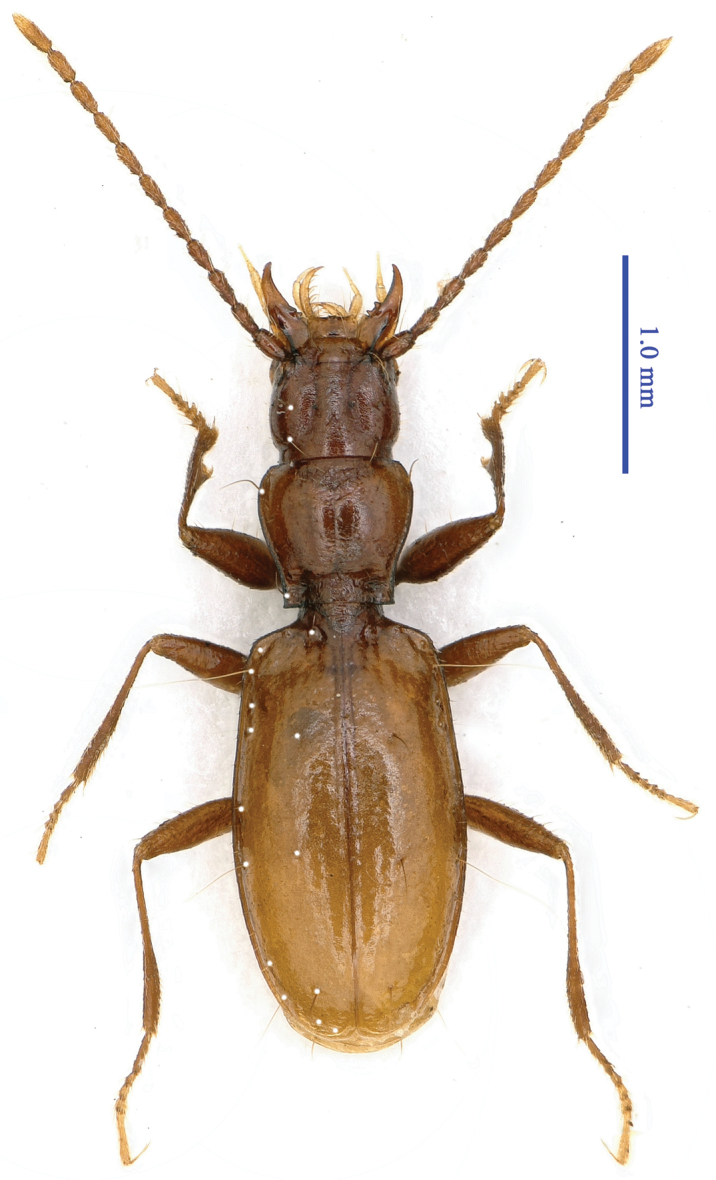
Habitus of *Wanoblemus wui* Tian & Fang, gen. n., sp. n., male, holotype.

Length: 3.9–4.0 mm, including mandibles (3.5–3.6 mm, excluding mandibles); width: 1.0–1.1 mm. Habitus as in Fig. 4.

Body brownish, palps and tarsi pale; surface sparsely pubescent, setae on pronotum longer on the underside of head, prosternum and propleura glabrous; legs densely pubescent, covered with long setae; micro sculptural engraved meshes nearly isodiametric, although irregular on head and pronotum moderately transverse on elytra, meshes well marked on head, but absent on elytra.

Head subquadrate, longer than wide, HLm/HW = 1.5, HLl/HW = 1.4; front and vertex moderately convex; frontal furrows fairly long and complete, nearly parallel medially; genae slightly expanded laterally; anterior supraorbital pores located in the middle of genae, sub-equidistant to lateral margin of genae and to posterior pore; clypeus 4-setose, labrum transverse, nearly straight in the front margin, 6-setose; mandibles distinctly curved at apex; ligula short, adnated with paraglossae, 6-setose at apex; palps short, penultimate joints much stouter than apical joints; labial palpomere 2 as long as labial palpomere 3, bisetose on inner margin, with two or three additional setae at the outer margin of the subapex; maxillary palpomeres 3 and 4 subequal in length; suborbital pores located on ventral side of genae; antennomeres subequal in length, except for antennomere 11, which is longer than the others.

Pronotum slightly transverse, PnL/PnW = 0.97, shorter than head, PnL/HLm = 0.76, slightly wider than head, PnW/HW = 1.19, lateral margin finely beaded, anterior lateromarginal pores located at about 1/5 of the apex; posterior lateromarginal pores just before hind angles; base narrower than front, PbW/PfW = 0.87, both nearly straight and unbeaded; frontal impression faint, basal transverse sulcus well-marked; disc strongly convex; scutellum small and short.

Elytra elongate, distinctly longer than fore body, EL/(HLm + PnL) = 1.27, EL/(HLl + PnL) = 1.5, much longer than wide, EL/EW = 1.77; elytra much wider than pronotum, EW/PnW = 1.53; base not bordered; disc moderately convex but fairly depressed near the base; basal pores on either side of scutellum, apical stria absent; anterior and posterior dorsal pores located on stria 3, at about 2/7 and 4/7 from the base of the elytra, respectively; pre-apical pores at about 1/8 of the apex of the elytra, closer to the suture than to the elytra margin; pores 1 and 2 of the marginal umbilicate series close to marginal gutter, pore 2 closer to pore 1 than to pore 3; pores 5 and 6 of middle group closely located; pore 10 near apical margin.

Legs moderately long, densely pubescent; protarsi short, tarsomere 1 shorter than tarsomeres 2 and 3 combined, tarsomeres 3 and 4 as long as wide; tarsomere 1 subequal to, or longer than, tarsomeres 2 to 4 combined in meso- and metatarsi; each abdominal ventrite IV–VI bearing a pair of paramedian setae.

**Figure 5.**
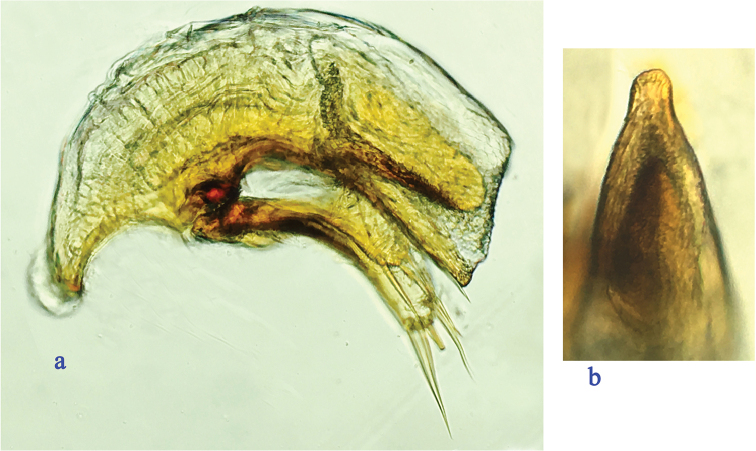
Male genitalia of *Wanoblemus wui* Tian & Fang, gen. n., sp. n. **a** median lobe and parameres, lateral view **b** apical lobe, dorsal view.

Male genitalia (Fig. 5): Median lobe of the aedeagus well-sclerotized, small but stout, strongly curved ventrally in the middle part, blunt at the apex; base fairly large, sagittal aileron very small and hyaline, inner sac with a large and thick copulatory piece, which is covered with scales, almost 1/3 the length of the aedeagus; in dorsal view, apical lobe short and broad; parameres elongate, right paramere longer than left paramere, each armed with three long setae at apex.

#### Etymology.

In honor of Yunhe Wu (College of Life Science, Anhui University, Hefei), an active collector of cave insects.

#### Distribution.

China (Anhui Province). Collected from a single limestone cave in Xuancheng Shi (Fig. 3). Baiyun Dong cave is approximately 1,000 m long and has a total area of about 20,000 m^2^. This show cave contains many impressive stalactites. Beetle specimens were sampled from the ground surface, in a dark zone covered with abundant litter and bat guano.

## Supplementary Material

XML Treatment for Cimmeritodes (Zhecimmerites) parvus

XML Treatment for Wanoblemus

XML Treatment for Wanoblemus wui
